# Immunometabolic Stress and Ketone Bodies in Disorders of the Aging Brain

**DOI:** 10.1093/geroni/igab046.1343

**Published:** 2021-12-17

**Authors:** John Newman

**Affiliations:** Buck Institute for Research on Aging, Novato, California, United States

## Abstract

Delirium is an acute confusional state that is a common complication of acute illness in older adults, and is associated with increased risk of death, disability, and dementia. Delirium in older adults is an example of a geriatric syndrome, with multifactorial, multi-system causes that include existing aging-related physiological changes as well as external acute stressors. Its pathophysiology delirium is not well understood but may include glycolytic energy deficits associated with acute inflammation in the brain. The endogenous ketogenic system provides ketone bodies as a lipid-derived alternative to glucose for cellular energy, and ketone bodies are increasingly understood to have immunomodulatory effects particularly on innate immune cells. We used a mouse model of acute inflammation-associated behavioral change to investigate how age-related differences in energy utilization in the brain affect delirium-like phenotypes, focusing on energy metabolism and innate immune activation in the brain as an example of immunometabolic approaches to geriatric syndromes.

